# Impact of processing and analysis methodology on thalamic susceptibility assessment in multiple sclerosis

**DOI:** 10.1371/journal.pone.0332478

**Published:** 2025-11-14

**Authors:** Fahad Salman, Niels Bergsland, Michael G. Dwyer, Jack A. Reeves, Abhisri Ramesh, Dejan Jakimovski, Bianca Weinstock-Guttman, Robert Zivadinov, Ferdinand Schweser

**Affiliations:** 1 Buffalo Neuroimaging Analysis Center, Department of Neurology at the Jacobs School of Medicine and Biomedical Sciences, University at Buffalo, The State University of New York, Buffalo, New York, United States of America; 2 Department of Biomedical Engineering, University at Buffalo, The State University of New York, Buffalo, New York, United States of America; 3 Center for Biomedical Imaging, Clinical and Translational Science Institute, University at Buffalo, The State University of New York, Buffalo, New York, United States of America; 4 Department of Imaging Sciences, Strong Memorial Hospital, University of Rochester, Rochester, New York, United States of America; 5 Jacobs Neurological Institute, Buffalo, New York, United States of America; University of Rochester, UNITED STATES OF AMERICA

## Abstract

**Background:**

Studies using quantitative susceptibility mapping (QSM) to investigate thalamic iron levels in people with multiple sclerosis (pwMS) have yielded inconsistent results. It has been speculated that cohort differences are responsible for these inconsistencies, leading to the phenomenological “early-rise late-decline” hypothesis, which posits that cohort age differences explain conflicting thalamic susceptibility findings. In a recent replication study, the authors failed to reproduce elevated thalamic susceptibility in pwMS previously reported by one of the only two QSM-based studies, despite matching cohort characteristics and processing, weakening the support for the phenomenological hypothesis.

**Objective:**

To investigate if the outcome of the recent replication study is robust with respect to different QSM algorithms and analysis methodologies.

**Methods:**

Using the same MRI dataset as the previous replication study, we assessed thalamic susceptibility across 83 pwMS and 44 healthy controls. To comprehensively evaluate methodological variability, we tested combinations of three background field removal (BFR) algorithms using various regularization parameters, four dipole inversion algorithms, three reference regions, and two segmentation methods. Each unique combination of a BFR algorithm (with its specific parameter) and a dipole inversion algorithm constituted a distinct pipeline, yielding a total of 19,558 susceptibility maps across 154 different pipelines.

**Results:**

Thalamic susceptibility was lower in pwMS compared to controls independent of the chosen methodology, with differences in effect sizes primarily driven by the background field removal algorithms and their regularization parameters, reference region, and segmentation method. The impact of dipole inversion algorithms was minimal.

**Conclusions:**

Our study suggests high reproducibility of group-level clinical studies using QSM to study the thalamus in pwMS. In particular, methodological differences in processing and analysis are unlikely to explain contradicting findings of thalamic susceptibility in MS.

## 1. Introduction

Iron is essential for normal brain function [1], supporting oxygen metabolism [2], myelination [3], and neurotransmitter synthesis [4], but its dysregulation contributes to multiple sclerosis (MS) pathogenesis [5–8]. In deep gray matter (DGM) structures, abnormal iron accumulation or depletion may drive neurotoxicity, oxidative stress, and neurodegeneration [4]. Quantitative susceptibility mapping (QSM), a technique exquisitely sensitive to paramagnetic and diamagnetic tissue components [9], has consistently demonstrated elevated iron levels throughout DGM structures in people with MS (pwMS) compared to healthy controls – with the notable exception of the thalamus [10,11].

The thalamus serves as a central hub for cognitive functions [12,13], and its atrophy has been recognized as an early and reliable marker of disease progression in MS [14–17]. As such, assessing thalamic iron levels may provide valuable insights into underlying pathophysiological mechanisms and help improve prognostic assessments. However, findings on thalamic iron levels in MS remain controversial. Most QSM- and R2*-based studies indicated decreased [18–26] or unchanged iron levels in pwMS [27–34]. In contrast, based on systematic reviews of QSM and R2* studies [10,11], only two studies—Rudko et al. [35] and Cobzas et al. [36]—have reported increased thalamic iron levels, highlighting the rarity of this finding. To reconcile these discrepancies, a phenomenological “early-rise late-decline” hypothesis was proposed, suggesting that thalamic iron levels increase early in the disease course in young individuals before decreasing in later stages in older individuals [24]. This hypothesis was supported by reports of increased thalamic susceptibility and R_2_* in younger pwMS (average ages below 40 years) [35,36] compared to controls, and decreased susceptibility and R_2_* in older pwMS (above 40 years) [18–26].

Salman et al. [37] recently replicated the 2014 study by Rudko et al., one of the two QSM studies reporting increased thalamic susceptibility in pwMS. Despite closely matching Rudko et al.‘s methodology, the study found decreased (not increased) thalamic susceptibility in pwMS, challenging the ‘early-rise’ hypothesis. Considering the notion that QSM outcomes may depend on methodological differences [38–49], the present study followed up on the replication study [37] with a comprehensive investigation of the robustness of the finding of reduced thalamic susceptibility. This study aimed to both confirm the robustness of the replication study’s findings and elucidate factors contributing to variability in QSM-based iron measurements. Using the same MRI data as the replication study (“younger cohort” therein), we systematically evaluated the impact of choosing different background field removal (BFR) algorithms [50–52], BFR algorithmic regularization parameters, dipole inversion algorithms [53–55], reference regions [35,38], and segmentation techniques [43,56] on thalamic susceptibility measurements.

## 2. Study design

To address the impractical computational cost of testing all possible combinations of QSM algorithms and analytical methods, we implemented a hierarchical approach. First, we reconstructed magnetic susceptibility maps using the same QSM algorithms as the replication study [37] (BFR = sophisticated harmonic artifact reduction for phase [SHARP] [50] and dipole inversion = morphology enabled dipole inversion [MEDI] [55]) and examined whether alternative analytical approaches—different segmentation methods and referencing regions—influenced group-level susceptibility findings. Subsequently, we investigated the impact of different BFR algorithms, their regularization parameters, and dipole inversion algorithms. To isolate algorithm-based effects, we retained the replication study’s segmentation method (FMRIB’s Integrated Registration and Segmentation Tool [FSL-FIRST] [56]) and referencing region (frontal deep white matter [FDWM]), ensuring any observed discrepancies would stem solely from the BFR and dipole inversion algorithm choices rather than analytical variations. All analyses performed in this study focused exclusively on the thalamus.

## 3. Methods

### 3.1. Subjects and MRI data acquisition

We used the exact same data as in the replication study by Salman et al. (Section 2.2 therein) [37]. In brief, the replication study had assembled a cohort that matched that in Rudko et al.’s study [35], referred to as *younger cohort* therein, using a large institutional database of scans collected in previous IRB-approved studies, with written informed consent obtained from all participants.. The cohort was designed to align with the group-level clinical and demographic characteristics reported in the original study by Rudko et al. All MRI data were collected between 2008-10-10 and 2019-02-07.

The resulting pwMS cohort had an average age (± standard deviation) of 37.4 ± 4.5 years and an Expanded Disability Status Scale (EDSS) of 1.7 (range: 0–6.0). The age- and sex-matched healthy control group (*N* = 44) had an average age of 36.9 (±4.5) years. Details are summarized in Table 1.

**Table 1:** Demographic and clinical characteristics of the younger cohort. M:F = Male:Female; CIS = Clinically Isolated Syndrome; RMS = Relapsing-Remitting Multiple Sclerosis; EDSS = Expanded Disability Status Scale.

Variables	Controls	pwMS
**Subjects**	44	83
**M:F Sex Ratio**	11:33	23:60
**Age in years** ^†^	36.9 ± 4.5	37.4 ± 4.5
**Disease Duration in years** ^†^	–	7.4 ± 6.6
**CIS:RMS ratio**	–	13/70
**EDSS** ^‡^	–	1.7 (0-6.0)

† Data are means ± standard deviations.

‡ Numbers in parentheses represent range of scores for all patients.

All scans were performed on the same 3T MRI scanner (Signa Excite HD 12.0; General Electric, Milwaukee, WI, USA) using an eight-channel head-and-neck coil, with no hardware or software upgrades over the acquisition period. Raw k-space data for QSM were acquired using an unaccelerated 3D single-echo spoiled gradient-recalled echo (SPGR) sequence with first-order flow compensation in the readout and slice directions. Acquisition parameters included a matrix size of 512 × 192 × 64, nominal resolution of 0.5 × 1 × 2 mm³ (FOV = 256 × 192 × 128 mm³), flip angle = 12°, TE/TR = 22/40 ms, bandwidth = 13.89 kHz, and total scan time of 8 minutes and 46 seconds. Additionally, a 3D T1-weighted (T1w) sequence was acquired using previously described parameters [21].

### 3.2. Analytical methods

The investigation of analytical methods used susceptibility maps reconstructed using SHARP [50] with a 7 mm kernel radius as the BFR and MEDI [55] as the dipole inversion with *N* = 7 regularization parameters (SHARP: λ_S_ [log-scaled range: 0.0057–0.0191] and MEDI: λ_D_ [250–4500]) that passed a visual quality assessment in the replication study [37] (acceptable pipelines therein), excluding pipelines (combinations of SHARP+MEDI parameters) with under- or over-regularized reconstructed maps. Specifically, a two-tiered visual quality assessment was performed: first, BF-corrected field maps were reviewed to identify and exclude BFR parameters yielding under- or over-regularized background field (BF)-corrected field maps; second, the final susceptibility maps were assessed to exclude inversion parameters that produced similarly suboptimal results.

#### 3.2.1. Thalamic segmentation.

We performed thalamic segmentation using FSL FIRST, following the methodology described in the replication study [37]. In addition, we used an advanced bi-parametric (QSM-T1w) multi-atlas segmentation technique optimized for QSM, Bi-parametric Joined Label Fusion (B-JLF; https://gitlab.com/R01NS114227/antsjointlabelfusion_biparametric). B-JLF utilizes nine QSM atlases for extreme ranges in age, volume, and DGM susceptibility to minimize segmentation biases [43]. We applied Advanced Normalization Tools (ANTs) joint label fusion technique using all atlases (*N* = 9) and propagated thalamic labels to the native subject spaces using subject-specific bi-modal warp field computations. The subject-space labels were then applied to all reconstructed susceptibility maps.

#### 3.2.2. Referencing region.

**Fig 1:**
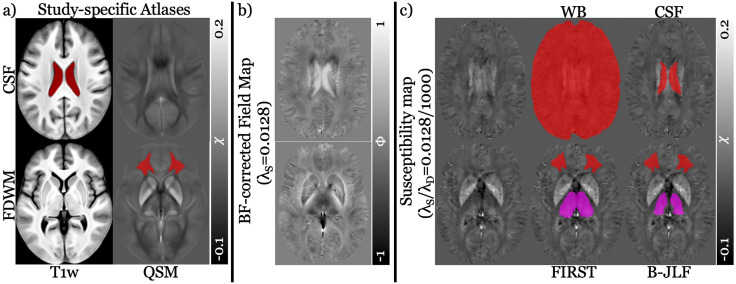
Template- and subject-Level QSM and T1w contrasts with regional segmentation overlays. a) T1-weighted (T1w) and quantitative susceptibility mapping (QSM) contrasts of the study-specific template generated using the bi-parametric approach. b) Representative subject’s background field (BF)-corrected field map from one of the SHARP parameters (λ_S_ = 0.0128) and c) susceptibility map from one of the SHARP+MEDI pipelines (λ_S_/λ_D_ = 0.0128/1000), in native space (41-year-old female, RMS patient [EDSS = 1.5]), with approximately matched slice views to those in (a). Top row: (a) and (c) display the manually delineated cerebrospinal fluid (CSF) region using the T1w contrast of the template (left column in a) and the subject-specific CSF label (right-most in c) obtained from the dual-contrast atlas in (a), respectively. Additionally, the whole brain (WB) mask (middle column in c) was obtained using the QSM pipeline, following the 2024 QSM Consensus Recommendations. Bottom row: (a) and (c) show the manually delineated frontal deep white matter (FDWM) region using the QSM contrast of the template (right column in a) and the subject-specific FDWM label (middle and right-most columns in c) segmented using the dual-contrast atlas in (a), respectively. Bi-lateral thalamic masks from FMRIB’s Integrated Registration and Segmentation Tool (FIRST) and Bi-parametric Joint Label Fusion (B-JLF) (violet) are displayed in the middle and right-most columns of (c), respectively, where mis-segmentation is evident in FIRST compared to B-JLF. The susceptibility template and map contrast range is in parts per million (ppm), while the field map contrast range is in radians (rad).

We first established a baseline by following the replication study’s referencing methodology – referencing bi-lateral susceptibility values from both segmentation methods (Section 3.2.1) to the FDWM region. Subsequently, we repeated the analyses using whole brain (WB) and cerebrospinal fluid (CSF) as alternative reference regions, selected for their frequent use in the literature [39,44], endorsement in recent QSM consensus guidelines [38], and practical advantages—WB offers largest voxel coverage, while CSF being away from the sinus cavity. The same FDWM mask was used as in the replication study (see *Referencing* in Section 2.4 therein) [37], while the WB and CSF masks were generated as described elsewhere [44]. All referencing masks are displayed in Fig 1a and c (bottom row: FDWM; top row: WB/CSF).

### 3.3. Voxel-wise analysis

Voxel-based statistics provide a detailed spatial assessment of susceptibility changes, reducing reliance on segmentation accuracy. To confirm that observed group differences were not biased by mis-segmentation, we conducted voxel-wise statistical analysis.

Each subject’s susceptibility maps from all pipelines were referenced to the FDWM (following the replication study) and spatially registered to the study-specific template. The normalized images were then analyzed using a nonparametric permutation-based analysis, as implemented in the FSL randomise program [57], with 10,000 permutations per analysis and age included as a covariate. The choice of 10,000 permutations was made to ensure the margin-of-error remained below 10% of the nominal alpha [57]. Threshold-free cluster enhancement was employed [58], and the analysis was restricted to the thalamus using a standard-space mask. This approach allowed us to determine group differences at *P* < 0.05, controlling for family-wise error rate. MRICron (v1.0.20201102) was used for the visualizations.

### 3.4. Additional QSM algorithms

We processed the data using two additional BFR algorithms alongside the SHARP BFR algorithm, as well as three additional dipole inversion algorithms (excluding MEDI).

We chose the BFR and inversion algorithms based on a recent study in which they demonstrated the highest reproducibility and sensitivity toward susceptibility changes over a period of 10 years across 6 BFR and 21 dipole inversion algorithms [44].

#### 3.4.1. Background field removal.

We applied SHARP using only the regularization parameters (λ_S_) discussed earlier (Section 3.2) and variable radius SHARP (VSHARP [50,51]) with a variable radius of 1:7 mm—within the optimal in-vivo range [59] and matching the SHARP’s fixed kernel to ensure comparability—across the full range of regularization parameters (including non-acceptable; λ_V_) as used for SHARP in the replication study (see *Background field removal* in Section 2.3 therein) [37], to further facilitate direct comparison. We also applied Regularization Enabled SHARP (RESHARP [52]) with a 3 mm kernel radius, using uniformly distributed 14 regularization parameter (λ_R_) values in log-scale, ranging from 0.0000001–0.1, and added 4th-order 3D polynomial fitting to suppress non-harmonic transceive phase contributions.

The RESHARP parameters were chosen to capture the full spectrum of BF-corrected field map appearances—including under-regularized (residual background fields), well-regularized (visually usable), and over-regularized (excessively smoothed) outputs—as was done for SHARP in the replication study and for VSHARP in this study. Refer to Table 2 for algorithm-specific regularization parameters utilized in this study.

**Table 2:** Regularization parameters (λ) used for the QSM algorithms. For the sophisticated harmonic artifact reduction for phase (SHARP) and morphology enabled dipole inversion (MEDI) algorithms, only the parameters deemed acceptable in the replication study are listed, which were used in this study. Variable radius SHARP (VSHARP); Regularized Enabled SHARP (RESHARP).

BFR Algorithm	Regularization Parameters
**SHARP (λ**_**S**_)	0.0057, 0.0074, 0.0085, 0.0104, 0.0128, 0.0156, 0.0191
**VSHARP (λ**_**V**_)	0.0031, 0.0038, 0.0046, 0.0057, 0.0074, 0.0085, 0.0104, 0.0128, 0.0156, 0.0191, 0.0234, 0.0287, 0.0351, 0.0430
**RESHARP (λ**_**R**_)	1.0e-7, 3.0e-7, 8.0e-7, 2.0e-6, 7.0e-6, 2.0e-5, 6.0e-5, 2.0e-4, 5.0e-4, 1.0e-3, 5.0e-3, 0.012, 0.035, 0.1
**Inversion Algorithm**	**Regularization Parameters**
**MEDI (λ**_**D**_)	250, 500, 750, 1000, 2000, 3000, 4500

We decided to adopt the same strategy as the replication study, as also described in Section 3.2 herein, limiting our analysis of the additional BFR algorithms to only the visually acceptable maps across all BFR-specific λ parameters (λ_V_ and λ_R_). The λ parameters were deemed acceptable after reconstructing all BF-corrected field maps and having two raters (F.Sa. and F.Sc.) visually inspect those from a randomly selected subset of subjects. During the visual inspection, the raters were blinded to the pipeline-specific group differences to ensure unbiased outcomes.

#### 3.4.2. Dipole inversion.

We applied Least Squares QR (LSQR [53]), Homogeneity Enabled Incremental Dipole Inversion (HEIDI [53]), and Approximate Message Passing with Parameter Estimation (AMPPE [54]). For LSQR and HEIDI, default parameters were used. For AMPPE, we used high-order (‘db1’) instead of the default low-order (‘db2’) wavelet bases its developer (personal communication with developer Shuai Huang from Emory University, Atlanta). We did not investigate the impact of different regularization parameters as observations with MEDI indicated that variations in regularization parameters (λ_D_) had a minimal impact on effect sizes [44](Fig 3 in the Replication Study) [37].

**Fig 2:**
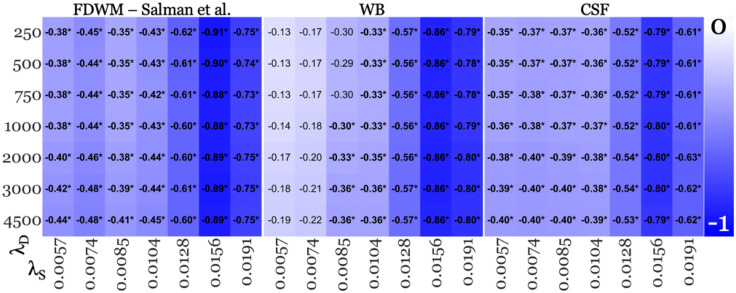
FIRST segmentation-based age-corrected effect sizes from SHARP+MEDI pipelines deemed acceptable in the replication study. Lower values (−1; blue – falsifies the “early rise” hypothesis) represent higher absolute negative effect size and white for effect size of 0. Each vertical panel corresponds to a reference region listed at the top, with the left-most panel using frontal deep white matter (FDWM) referencing as in the replication study, middle using whole brain (WB), and right-most using cerebrospinal fluid (CSF) referencing. Within each panel, each row corresponds to a regularization parameter value used for MEDI inversion algorithm (λ_D_, listed on the left-hand side), while each column represents a regularization parameter value used for SHARP BFR algorithm (λ_S_, listed at the bottom). Significant group differences were marked with asterisks (*).

Subject-specific masks utilized for the above algorithms were generated and applied in compliance with the 2024 QSM Consensus Recommendations [38], as described in the replication study.

### 3.5. QSM statistical analysis

Thalamic susceptibility values for each subject were calculated using FSLstats [60] with bi-lateral binary masks in native QSM image space, obtained from each segmentation method (only FIRST for additional algorithms in Section 3.4). Subsequently, susceptibility values from each segmentation method were referenced to each of the reference regions (only FDWM for Section 3.4).

Following the replication study’s protocols, we then 1) averaged bi-lateral referenced thalamic susceptibility values, 2) applied age correction, and 3) conducted independent t-tests for group comparisons. Subsequently, we calculated Cohen’s *d* effect sizes across pipelines to quantify the impact of parameter choices on study outcomes and visualized them using heat maps (Python Seaborn v0.12.2) [61].

Finally, median absolute effect sizes were calculated for all methods (referencing region, segmentation method, BFR algorithms, and inversion algorithms) using only the significant effect sizes (*P* ≤ 0.05). Following this, the percentage difference of median absolute effect sizes was calculated for all new methods used in this study, relative to the originally used methods in the replication study (BFR = SHARP, inversion = MEDI, segmentation = FIRST, and reference region = FDWM).

### 3.6. Scientific rigor

All processing and analyses were fully automated to ensure consistency and reproducibility, as described in the replication study. Specifically, the QSM processing was fully automated and executed in a standardized computational environment using containerized computing on a high-performance cluster [62]. Additionally, subject-specific QSM-based montages were generated for inspection, comprising both BF-corrected field maps (without regional overlays) and susceptibility maps (with and without thalamus, FDWM, WB, and CSF overlays). These montages were carefully reviewed by an experienced QSM user (F.Sa., 6.5 years of neuroimaging experience) to confirm the absence of artifacts (e.g., Gibbs ringing, motion, and/or transceive phase inhomogeneities). Scans were excluded if DGM regions were substantially affected by such artifacts. These montages also served to verify accurate placement of segmentation masks on native susceptibility maps.

A total of 19,558 susceptibility maps were reconstructed in this study: 6,223 maps for Section 3.2 (127 subjects × [7 λ_S_ × 7 λ_D_]) and 13,335 maps for Section 3.4 (127 subjects x [7 λ_S_ + 14 λ_V_ + 14 λ_R_] × 3 dipole inversion algorithms). These susceptibility maps underwent 50,673 total analyses: 37,338 in Section 3.2 (6,223 maps × 2 segmentation methods × 3 reference regions) and 13,335 in Section 3.4 (13,335 maps × 1 segmentation method × 1 reference region).

## 4. Results

### 4.1. Quality control

Figs 1b and c displays a representative native-space BF-corrected field map and susceptibility map reconstructed using the SHARP (λ_S_ = 0.0128) + MEDI pipeline (λ_S_/λ_D_ = 0.0128/1000), respectively, along with all corresponding segmented regional masks overlayed on the susceptibility map (middle and right-most columns in Fig 1c) – including the thalamic region of interest (ROI) derived from both FIRST and B-JLF segmentation methods. Quality assessment revealed no artifacts in either the BF-corrected field maps (Fig 1b) or susceptibility maps (left-most column in Fig 1c); therefore, no subjects were excluded from the study.

The middle and right-most columns in Fig 1c demonstrate subject-specific mask placement for all ROIs (thalamus, FDWM, WB, and CSF). However, clear thalamic mis-segmentation was observed from FIRST (bottom middle panel in Fig 1c), extending into neighboring white matter (WM) region, specifically into the internal capsule, as observed previously [40,63]. Conversely, B-JLF did not extend outside the thalamic boundary (bottom right-most panel in Fig 1c). Importantly, none of the subjects’ thalamic masks from either FIRST or B-JLF, nor any of the reference region masks, showed incorrect placement (i.e., not centered on the intended regions or shifted).

### 4.2. Findings across referencing regions and segmentation methods

**Fig 3:**
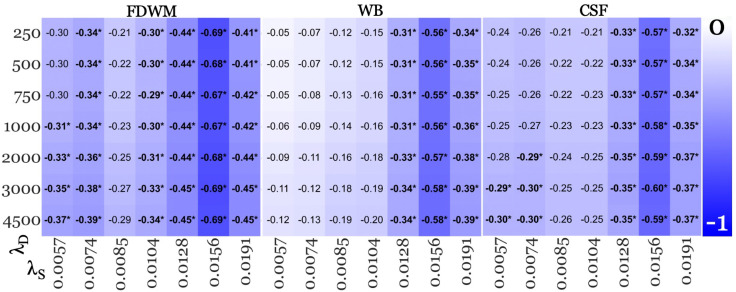
B-JLF segmentation-based age-corrected effect sizes from SHARP+MEDI pipelines deemed acceptable in the replication study. Lower values (−1; blue – falsifies the “early rise” hypothesis) represent higher absolute negative effect size and white for effect size of 0. Each vertical panel corresponds to a reference region listed at the top (frontal deep white matter [FDWM]; whole brain [WB]; cerebrospinal fluid [CSF]). Within each panel, each row corresponds to a regularization parameter value used for MEDI inversion algorithm (λ_D_, listed on the left-hand side), while each column represents a regularization parameter value used for SHARP BFR algorithm (λ_S_, listed at the bottom). Significant findings were marked with asterisks (*).

**Fig 4:**
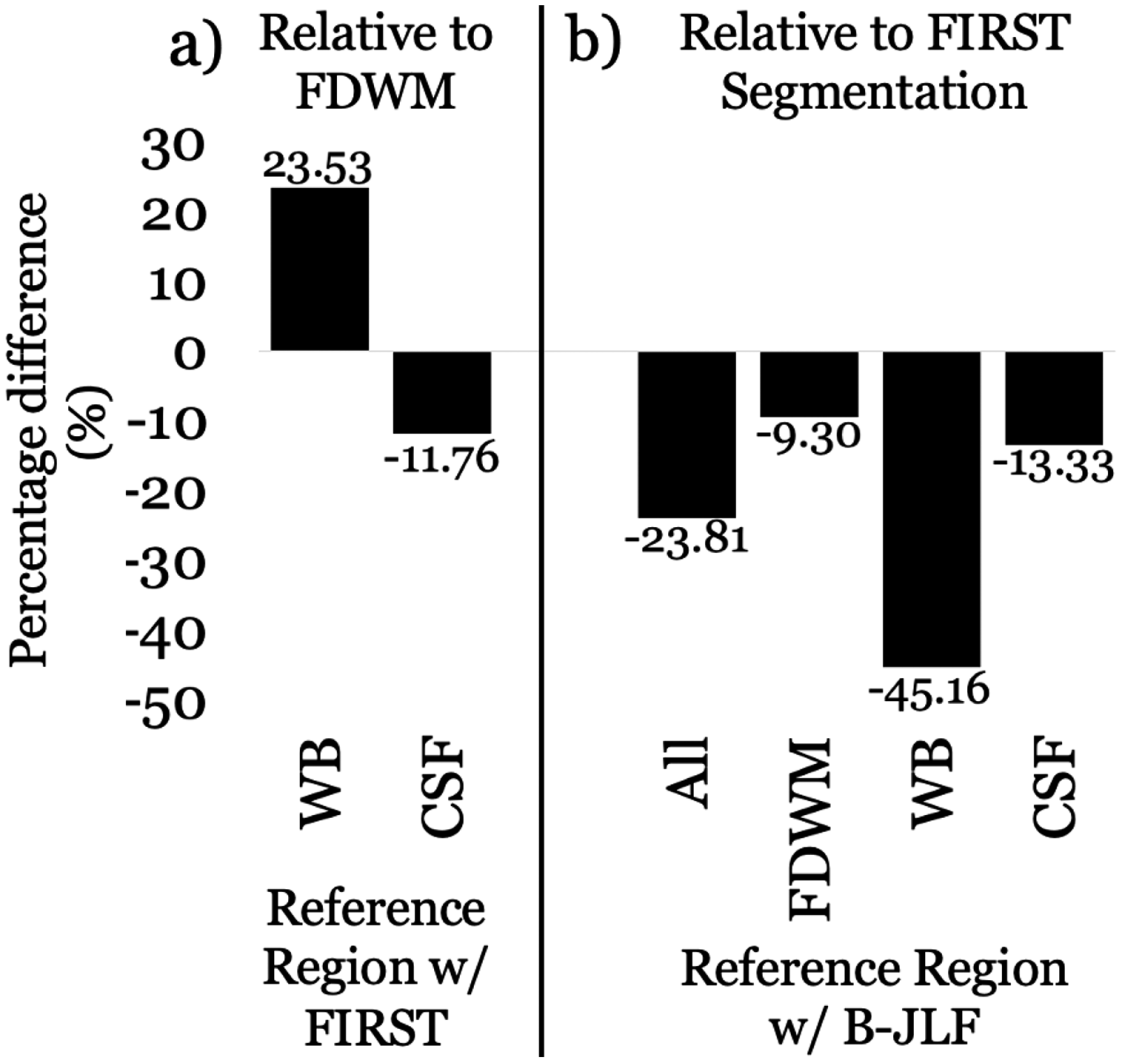
For SHARP+MEDI pipelines, median percentage difference in effect sizes from (a) different reference regions with FMRIB’s Integrated Registration and Segmentation Tool (FIRST) segmentation, relative to the originally used frontal deep white matter (FDWM) reference region, and (b) the Bi-parametric Joint Label Fusion (B-JLF) segmentation method, both across all reference regions (All) and individually for each reference region (FDWM, whole brain [WB], and cerebrospinal fluid [CSF]), relative to FIRST and their respective counterparts. Median percentage differences are indicated outside the bars, with negative values indicating a decrease in absolute effect sizes and positive values indicating an increase.

Figs 2 and 3 summarize the thalamic effect sizes from each pipeline across different reference regions and the two segmentation methods, respectively. To summarize the impact of reference regions and segmentation methods on effect sizes, Fig 4 illustrates the median percentage difference in absolute effect sizes for (a) WB and CSF referencing relative to FDWM with FIRST segmentation, and (b) B-JLF segmentation with each reference region compared to FIRST segmentation and the corresponding reference region.

#### 4.2.1. Initial thalamic findings in pwMS - replication study [37].

Thalamic susceptibility was consistently lower in patients than in controls, with effect sizes ranging from −0.35 to −0.91 (min and max variation: 117.07% and 160.00% variation with λ_D_ = 4500 and 250, respectively, across λ_S_ values). These findings are summarized in the left-most panel of Fig 2 (FDWM referencing).

#### 4.2.2. Effect of changing the referencing region.

Changing the reference regions from FDWM to WB and CSF did not affect the overall findings of lower thalamic susceptibility in patients, as shown in the middle and right-most panels of Figs 2 and 3, respectively.

The absolute effect sizes were 23.53% higher (median difference) with WB referencing and 11.76% lower with CSF referencing, compared to FDWM referencing, as shown in Fig 4a. Notably, with all reference regions, the highest absolute effect sizes were observed at the higher end of the acceptable range for λ_S_, with λ_S_ = 0.0156 yielding the largest effect sizes, ranging from −0.88 to −0.91 with FDWM, −0.86 with WB, and −0.79 to −0.80 with CSF referencing, across λ_D_ values (Fig 2).

#### 4.2.3. Effect of the segmentation method.

As shown in Fig 4b, compared to the FIRST segmentation outcomes, B-JLF segmentation resulted in 23.81% lower effect sizes (median difference) across all reference regions, with the largest difference observed with WB referencing (−45.16%), followed by minor differences with CSF (−13.30%) and FDWM referencing (−9.30%).

While the choice of segmentation method substantially affected the magnitude of effect sizes, it did not alter the qualitative outcomes, i.e., the declining trend remained unaffected. Additionally, similar comparability in effect sizes was observed across reference regions with B-JLF segmentation (Fig 3), as with FIRST.

### 4.3. Voxel-wise analysis

**Fig 5:**
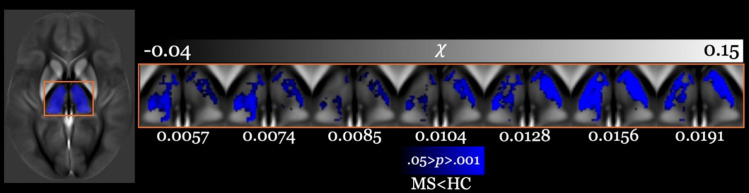
Voxel-wise analysis of susceptibility maps using the same slice (chosen for the best thalamic visuals) of the study-specific (QSM contrast) template shown in bottom row of Fig 1a, referenced to the FDWM from acceptable λ_S_ values (listed at the bottom) and MEDI λ_D_ = 1000 within the thalamus comparing all multiple sclerosis (MS) patients to healthy controls (HC). Results were corrected for age and are shown at *P* < 0.05, corrected for family-wise error rate. Areas of significantly lower susceptibility in patients compared with HCs are shown in blue. Darker shades are indicative of smaller *P* values. The contrast range is in part-per-million (ppm).

Fig 5 displays FDWM-referenced voxel-wise analysis from all λ_S_ values with λ_D_ = 1000 (default for MEDI), focusing on the thalamic region using the B-JLF segmented mask due to its superior segmentation performance compared to FIRST segmentation, as discussed in Section 4.1.

Voxel-wise analysis revealed significant clusters of reduced thalamic susceptibility in pwMS compared to controls, with the most prominent differences localized to the lateral thalamic nucleus. All voxel-wise visuals corroborated our B-JLF-specific FDWM-referenced group differences (Fig 3 – left-most panel).

### 4.4. Effect of BFR and inversion algorithm

#### 4.4.1. Visual inspection of field and susceptibility maps.

**Fig 6:**
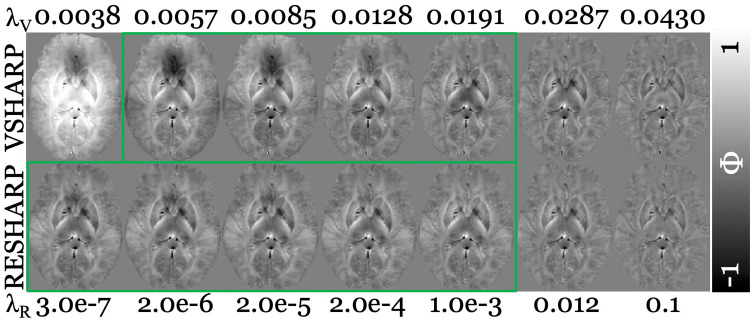
Background field-corrected field maps in native space of a representative subject (38 years old female; RMS patient [EDSS = 0]) from additional BFR algorithms (listed on the left-hand side; Variable radius [V] Sophisticated Harmonic Artifact Reduction for Phase [SHARP; VSHARP] and Regularized Enabled SHARP [RESHARP], respectively) over a range of alternative λ values (listed at the top [λ_V_] and bottom [λ_R_], respectively). Green boxes indicate acceptable λ values for both BFR algorithms, respectively. Contrast range is in radians (rad). See Fig 7 for susceptibility maps from AMPPE, LSQR, and HEIDI inversion algorithms across all three BFR algorithms (select regularization parameter).

Fig 6 illustrates the absence of artifacts in the BF-corrected field maps generated using VSHARP and RESHARP across various λ_V_ and λ_R_ values, respectively. However, clear signs of under- and over-regularization were observed within the field maps from VSHARP, particularly with λ_V_ values of 0.0038 and lower (assuming this pattern persists at lower values), and 0.0287–0.0430. In contrast, RESHARP’s tolerance level led to the results being stagnant around λ_R_ = 2.0e-5 and lower, without overly under-regularized solutions.

Visually acceptable appearances of field maps (green box) were observed with λ_V_ values between 0.0057 and 0.0191 and λ_R_ values of 1.0e-3 and lower, hence, the subsequent analysis was limited to the acceptable parameter ranges.

**Fig 7:**
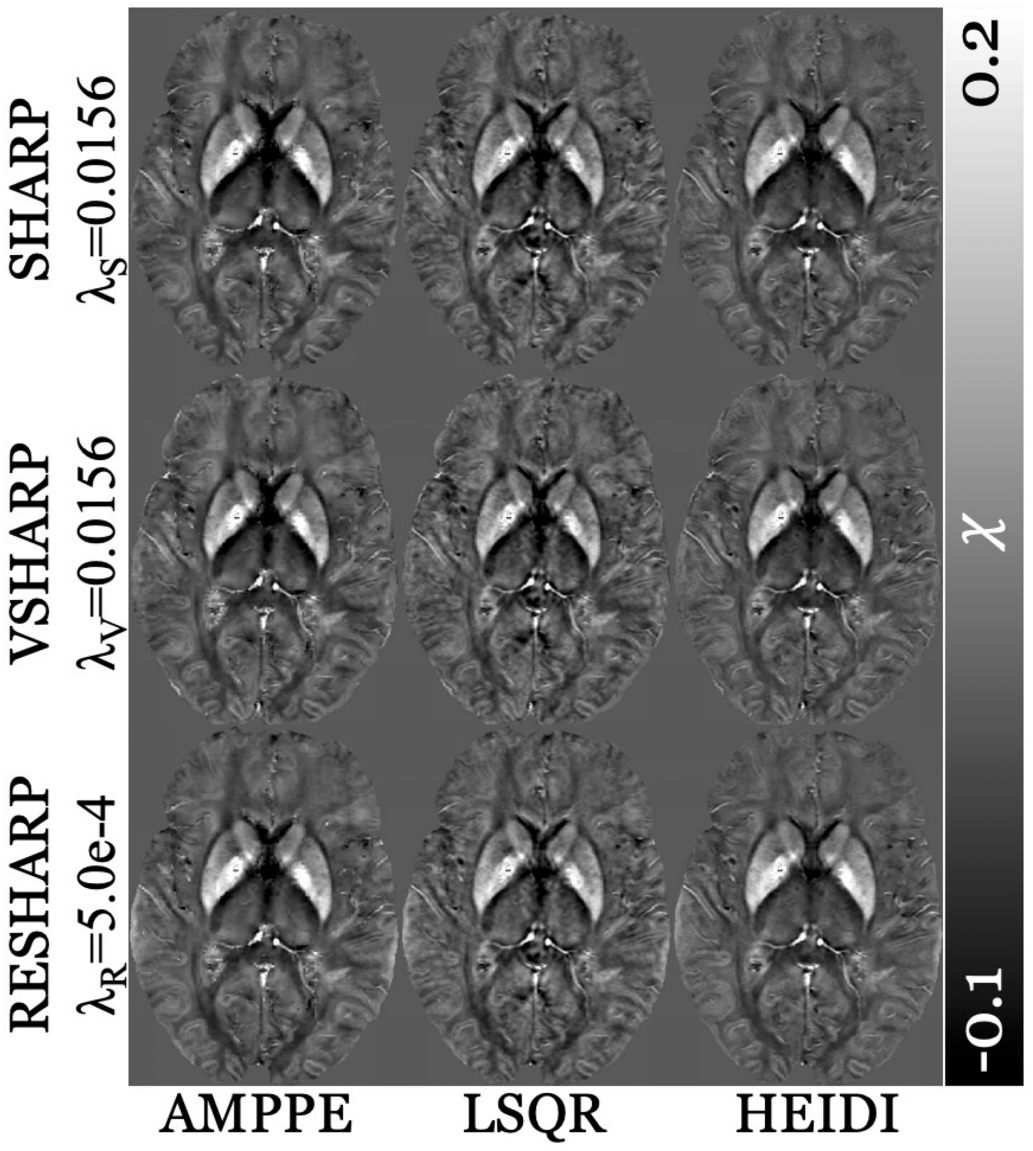
Susceptibility maps in native space of a representative subject (38 years old female; RMS patient [EDSS = 0]) from additional inversion algorithms (listed at the bottom; Approximate Message Passing with Parameter Estimation [AMPPE], Least Squares QR [LSQR], Homogeneity Enabled Incremental Dipole Inversion [HEIDI], respectively) across all BFR algorithms (listed on the left-hand side with their respective regularization parameters [λ]; Sophisticated Harmonic Artifact Reduction for Phase [SHARP, λ_S_]; Variable radius SHARP [VSHARP, λ_V_] and Regularized Enabled SHARP [RESHARP, λ_R_], respectively). Contrast range is in part-per-million (ppm).

Fig 7 displays the susceptibility maps reconstructed using inversion algorithms with specific BFR algorithmic λ values. Susceptibility maps were visually comparable across all algorithmic combinations (BFR+inversion), with no artifacts observed in any reconstruction.

#### 4.4.2. No effect on qualitative outcome.

**Fig 8:**
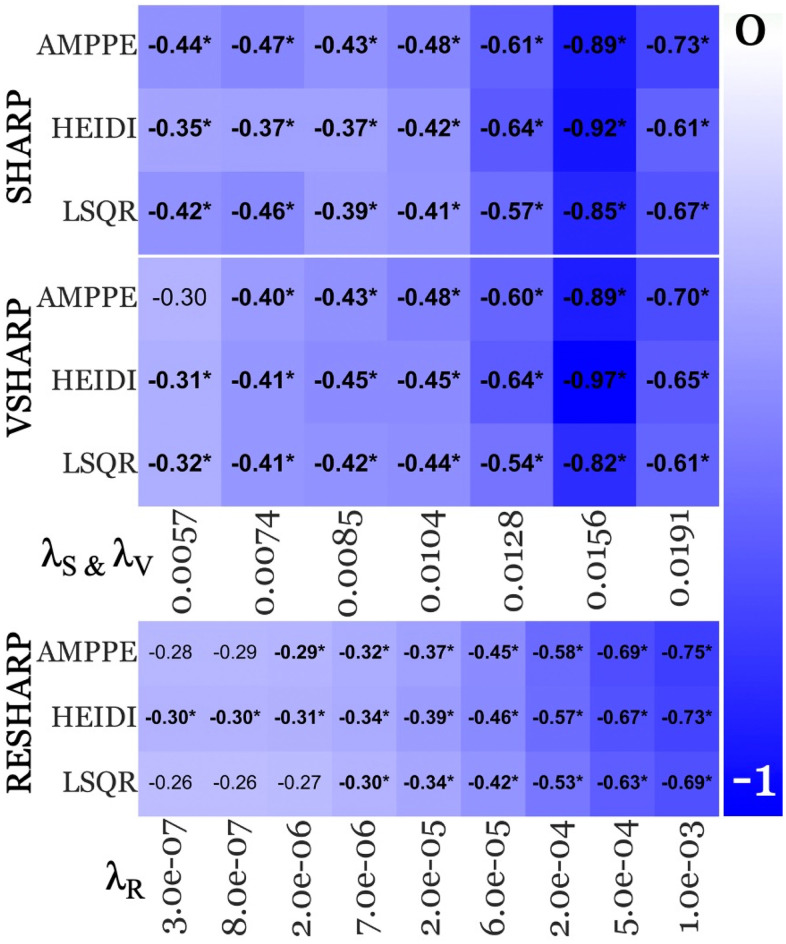
FIRST segmentation FDWM-referenced age-corrected effect sizes across BFR-specific regularization parameters with three inversion algorithms. Each horizontal panel portrays the results of one specific BFR algorithm that is denoted at the left-hand side of the panel (Sophisticated Harmonic Artifact Reduction for Phase [SHARP]; Variable radius SHARP [VSHARP] and Regularized Enabled SHARP [RESHARP], respectively). Within each panel, each row corresponds to a specific inversion algorithm (listed on the left-hand side; Approximate Message Passing with Parameter Estimation [AMPPE], Least Squares QR [LSQR], Homogeneity Enabled Incremental Dipole Inversion [HEIDI], respectively), while each column represents an acceptable BFR-specific regularization parameter listed at the bottom of the horizontal panel (RESHARP = λ_R_; same for SHARP [λ_S_] and VSHARP [λ_V_]– see top row in Fig 6). Lower values (−1; blue – falsifies our “early rise” hypothesis) represent higher absolute negative effect size and white for effect size of 0.

Fig 8 portrays FDWM-referenced FIRST-segmented thalamic effect sizes from all BFR algorithms and three inversion algorithms (AMPPE, HEIDI, and LSQR). The trend of lower susceptibility in pwMS compared to the control group, what was observed previously in the replication study (using SHARP+MEDI) [37], remained unchanged regardless of the choice of the BFR algorithm, its parameter, or the inversion algorithm, with only the magnitude of effect sizes being affected.

#### 4.4.3. Variable λ ranges for SHARP-based BFR algorithms with higher effect sizes.

Each inversion algorithm exhibited a similar trend between SHARP (top panel in Fig 8) and VSHARP (middle panel), with λ_S_ and λ_V_ = 0.156 yielding the highest effect sizes, consistent with the SHARP+MEDI pipelines (Figs 2 and 3; see Section 4.2). As for RESHARP (bottom), the highest effect size was observed at λ_R_ = 0.001 (AMPPE = −0.75, LSQR = −0.69, HEIDI = −0.73; *P* < 0.05).

In summary, effect sizes varied by 162.86% with SHARP (range: −0.35 to −0.92 across all inversion algorithms), 212.90% with VSHARP (−0.31 to −0.97), and 150.00% with RESHARP (−0.30 to −0.75), with the lowest effect size observed with the RESHARP+HEIDI/LSQR pipeline (−0.30; *P* < 0.05) and the highest with the VSHARP+HEIDI pipeline (−0.97; *P* < 0.05). Among all inversion algorithms across BFR methods, HEIDI was the most robust inversion method, yielding the highest number of significant effect sizes (*N* = 23).

#### 4.4.4. Outcomes compared to the originally implemented algorithms.

**Fig 9:**
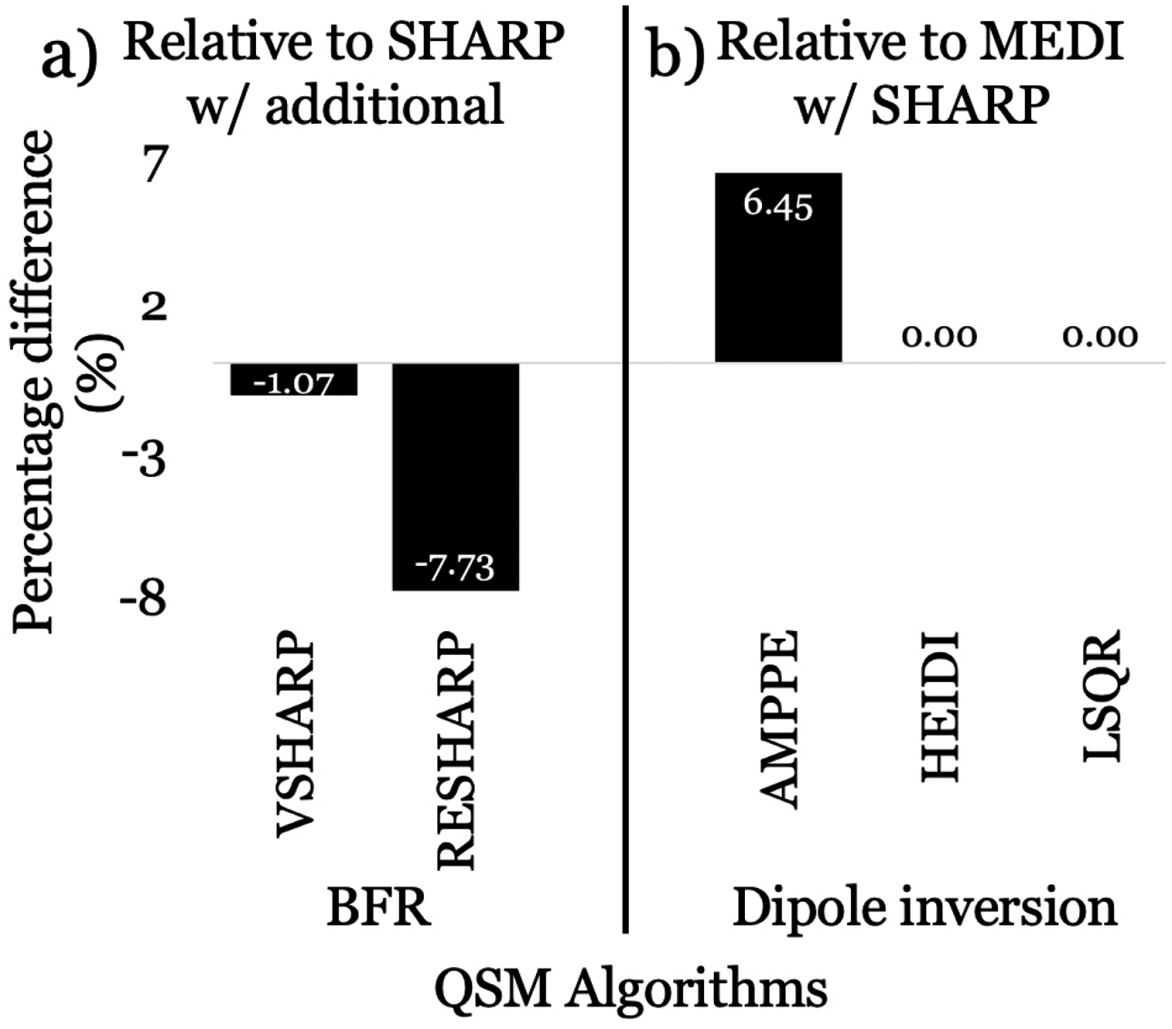
Median percentage difference in absolute effect sizes due to variations in quantitative susceptibility mapping (QSM) algorithms. (a) Effect size percentage differences for Variable radius (V) Sophisticated Harmonic Artifact Reduction for Phase (SHARP; VSHARP) and Regularized Enabled SHARP (RESHARP) relative to SHARP, calculated using the median effect sizes across all three additional dipole inversion algorithms (Approximate Message Passing with Parameter Estimation [AMPPE], Homogeneity Enabled Incremental Dipole Inversion [HEIDI], and Least Squares QR [LSQR]) for each background field removal (BFR) algorithm. (b) Effect size percentage differences for AMPPE, HEIDI, and LSQR relative to morphology enabled dipole inversion (MEDI). Since MEDI was only used with SHARP, the median effect size for each additional inversion algorithm was computed across SHARP and compared to SHARP+MEDI. Median percentage differences are indicated inside the bars, with negative values indicating a decrease in absolute effect sizes and positive values indicating an increase.

Fig 9 plots the median percentage difference in absolute effect sizes from algorithms utilized for this sub-analysis (BFR = SHARP, VSHARP, RESHARP – Fig 9a; inversion = AMPPE, HEIDI, and LSQR – Fig 9b) compared to the ones from the replication study (SHARP and MEDI), using the originally used analytical methods (FIRST segmentation and FDWM referencing).

Focusing on the BFR algorithms, VSHARP portrayed 1.07% lower effect sizes, while RESHARP portrayed 7.73% lower effect sizes compared to SHARP across all inversion algorithms within this sub-analysis. On the other hand, for inversion algorithms with the SHARP BFR algorithm, AMPPE exhibited 6.45% higher effect sizes compared to MEDI, while HEIDI and LSQR showed no differences relative to MEDI (0%).

Notably, the findings of the current sub-analysis align with those of the replication study, where the BFR algorithm’s regularization parameters had a substantially greater impact on effect sizes (160.00% variation) than the choice of inversion algorithm (or its parameter; ≤ 15%).

## 5. Discussion

### 5.1. Study outcome

This study builds on Salman et al.’s recent replication work, which tested the “early-rise late-decline” hypothesis and failed to reproduce findings of increased thalamic susceptibility in a relatively young cohort, refuting the “early-rise” aspect. The results of the present study demonstrated remarkable robustness of reduced thalamic susceptibility findings in pwMS compared to healthy subjects across diverse QSM processing and analytical approaches. Consequently, differences in processing methods, their parameters, or analysis routines are unlikely to explain the heterogeneity of thalamic susceptibility findings in the literature.

These findings strengthen the potential of thalamic susceptibility as an imaging biomarker for MS-related neurodegeneration. Given the thalamus’ early involvement in MS and its association with cognitive decline and disability progression [16,64], reliable detection of altered susceptibility could support earlier and more accurate disease staging, monitoring, and treatment response evaluation.

### 5.2. Consistent trends but variable effect sizes across analytical methods

We observed that the choice of reference region (FDWM to WB=+23.53% and CSF = −11.76% median difference; Fig 4a) and segmentation method (FIRST to B-JLF = −23.81%; Fig 4b) substantially influenced the effect size magnitude.

FIRST’s performance is critically dependent on anatomical contrast [49,65], which is particularly problematic in the thalamus where contrast is suboptimal on T1-weighted images. Recent studies indicated poor QSM-based DGM segmentation from FSL FIRST, which were shown to lead to biased susceptibility findings in pwMS, especially in the thalamus, where atrophy begins from the earliest stages of MS [40,63].

The higher absolute effect sizes observed with FIRST may stem from systematic mis-segmentation, where labels extended into neighboring white matter (WM) regions such as the internal capsule (Fig 1b, right-side section’s bottom middle panel), consistent with previous observations [40]. Notably, both this mis-segmented region (e.g., internal capsule) and the FDWM are known to be affected by MS pathology [38,39]. When used in demyelinating disorders, these compromised regions may artificially elevate thalamic susceptibility measurements in MS patients, consequently amplifying observed group differences and effect sizes [40,63]. This could explain why effect sizes were not only higher with FIRST segmentation but also with FDWM referencing compared to WB and CSF referencing.

### 5.3. Consistent qualitative study outcomes across QSM processing

Contrary to the notion that QSM-based qualitative study outcomes (such as significantly decreased thalamic susceptibility in pwMS) are dependent on parameter settings and algorithmic choices [41–43,48], thalamic outcomes (Figs 2, 3 and 8) were highly consistent across regularization parameters of the different BFR algorithms and dipole inversion algorithm in the present study. Moreover, thalamic outcomes remained robust even across dipole inversion algorithms.

### 5.4. BFR algorithm is a major source of effect size variation

Our findings indicate that BFR regularization parameters contributed to the highest variation in effect sizes, with a minimum variation of 102.56% across λ_S_ values (λ_D_ = 4500 FIRST/B-JLF + CSF; Figs 2 and 3 [significant findings]). This surpassed the variation observed due to the dipole inversion parameters (MEDI), which reached a maximum of 20.00% across λ_D_ values (λ_S_ = 0.0085 with FIRST+WB; Fig 2), segmentation methods, which showed a maximum variation of 56.96% when comparing FIRST to B-JLF at λ_S_/λ_D_ = 0.191/250 pipeline (WB in Figs 2 and 3), and reference regions, where the largest variation was 29.55% between FDWM and WB at λ_S_/λ_D_ = 0.128/1000 pipeline (B-JLF; Fig 3).

Moreover, in line with the replication study [37] and another recent study [44], effect sizes—likely influenced by differences in variance [45]—were primarily affected by the BFR algorithms (SHARP to VSHARP: −1.07%; RESHARP: −7.73%; Figs 8 and 9a) rather than the dipole inversion algorithms (MEDI to AMPPE: + 6.45%; HEIDI and LSQR: 0% median difference; Fig 9b). In summary, the BFR algorithms and their regularization parameters play the most critical role in group difference detection. Consequently, greater emphasis should be placed on optimizing BFR algorithms and their parameters over dipole inversion algorithms [46,47].

### 5.5. Toward QSM pipeline standardization

While our findings emphasize the substantial impact of QSM pipeline choices on susceptibility values, full standardization remains challenging. The main concern is not variation in effect size or noise, but rather discrepancies in absolute susceptibility values across pipelines, which undermine reproducibility and cross-study comparability. Based on our results and prior studies [38,44,66], we recommend using SHARP-based BFR methods—especially RESHARP—paired with a stable dipole inversion method such as HEIDI. Parameter selection should prioritize visually acceptable map quality, especially in settings where full numerical optimization of effect sizes is impractical due to time or computational constraints. Although we do not recommend a single standardized pipeline, clear reporting of all methodological components and transparent rationale for their selection are essential steps toward harmonization. Long-term efforts should focus on minimizing cross-study differences in absolute values (excluding acquisition-related noise), potentially through consensus protocols or phantom-based calibration.

### 5.6. Limitations

Although we did not explicitly investigate thalamic sub-nuclei, our additional voxel-wise analysis revealed susceptibility changes primarily in non-pulvinar regions (Fig 5), partially complementary to previous reports by Rudko et al. and Cobzas et al., which demonstrated R_2_*-based voxel-wise differences in the pulvinar, but not in lateral regions. These observations contribute to a nuanced understanding of the heterogeneity of intra-thalamic iron changes in pwMS [24,28,67]. It is possible that differences in nucleus-specific atrophy rate between study cohorts may have contributed to differences in QSM findings between cohorts [41,42,68–71]. Furthermore, future research should assess whether different QSM reconstruction and analytical methods yield consistent results in other brain regions.

While the use of a single-echo acquisition—as opposed to the multi-echo approach used by Rudko et al.—may result in susceptibility underestimation due to increased sensitivity to noise and artifacts [72,73], this bias is likely systematic and consistent across groups, as supported by Rua et al.’s supplementary materials (mmc1.xlsx) [73]. As such, it would primarily affect the absolute susceptibility values at the individual level without altering group differences or their direction. Nonetheless, we acknowledge this as a potential limitation and encourage further investigation.

We cannot exclude the possibility that our additional QSM reconstruction-based outcomes (Section 4.4) may have been influenced by not employing different regularization parameters for additional dipole inversion algorithms (Section 3.4.2). Although we observed minimal variation in effect sizes across MEDI parameters (maximum variation: 20%), each algorithm has unique nuances that could impact susceptibility estimates. Therefore, further investigation is warranted to determine whether different regularization choices for other dipole inversion algorithms might yield varying outcomes.

## 6. Conclusion

Despite employing alternative QSM reconstruction and analytical methods, we consistently observed reduced thalamic susceptibility in young pwMS. Our results highlight the critical role of BFR algorithms and their parameters in obtaining high effect sizes, underscoring the need for their careful optimization in QSM studies. Furthermore, our study suggests that QSM is a highly robust and reproducible technique for clinical research, supporting the comparability of published studies conducted with different reconstruction and analysis methodologies. This reinforces QSM’s potential as a reliable quantitative technique for investigating tissue susceptibility changes in neurological disorders.
